# The Treatment of Cleidocranial Dysostosis (Scheuthauer-Marie-Sainton Syndrome), a Rare Form of Skeletal Dysplasia, Accompanied by Spinal Deformities: A Review of the Literature and Two Case Reports

**DOI:** 10.1155/2018/4635761

**Published:** 2018-07-09

**Authors:** Mehmet Bülent Balioğlu, Deniz Kargın, Akif Albayrak, Yunus Atıcı

**Affiliations:** ^1^Department of Orthopaedics, Istinye University Liv Hospital, Istanbul, Turkey; ^2^Department of Orthopedics, Health Science University Baltalimani Bone Diseases Education and Research Hospital, Istanbul, Turkey; ^3^Department of Orthopaedics, Okan University Hospital, Istanbul, Turkey

## Abstract

Cleidocranial dysostosis is a skeletal dysplasia inherited in an autosomal dominant manner and may lead to complications such as scoliosis and kyphosis, concurrent with various orthopedic involvements. Since concurrent spinal deformities are of progressive nature, surgical treatment may be necessary. In addition to other orthopedic problems, possible accompanying complications such as atlanto-axial subluxation, myelopathy, syringomyelia, congenital spine deformities, spondylosis, and spondylolisthesis should be kept in mind while planning for the treatment of scoliosis and kyphosis. Lengthening the use of growth-friendly systems (growing rod) in patients, like ours, with an early onset of symptoms, and performing posterior instrumentation and fusion once the spinal growth is complete will yield successful results with no complications in the middle and the long term. Further multicenter studies with more comprehensive assessments are required to find solutions to spinal problems related to this rare skeletal dysplasia.

## 1. Introduction

Cleidocranial dysostosis is a skeletal dysplasia inherited in an autosomal dominant manner and is characterized by intramembranous bone formation. It causes abnormalities in the clavicle, cranium, and pelvis. The disorder was first described by Marie and Sainton in 1898 [1, 2] and is also known as cleidocranial dysplasia, Scheuthauer-Marie-Sainton syndrome, mutational dysostosis, osteodental dysplasia, generalized dysostosis, pelvicocleidocranial dysplasia, and cleidocranial-pubic dysostosis [3].

Cleidocranial dysostosis is a condition inherited in an autosomal dominant manner in which 1/3 of the patients show spontaneous mutation and 2/3 show familial variation [4]. The responsible gene RUNX2 (Runt-related transcription factor 2) is a cloned gene located on the short arm of Chromosome 6 (6p21) [5–8]. RUNX2 activates osteoblast differentiation as an osteoblastic-specific transcription factor and a regulator of osteoblast differentiation [6, 9]. It also controls the differentiation of precursor cells in osteoblasts. The cells secrete bone matrix and thus form a bone. In addition, RUNX2 plays a key role in the regulation of chondrocyte differentiation during endochondral bone formation. This new “principal gene” may explain the underlying mechanisms of bone formation in addition to the pathobiology of cleidocranial dysostosis [10].

Characteristic findings of cleidocranial dysostosis include hypoplasia or the absence of the clavicle, brachycephalic skull, hypoplasia in the middle of the face, delayed closure of the fontanelles, and slight to moderate shortness in stature. Although the most important abnormalities are seen in bone reformations through intramembranous ossification in the clavicle, cranium, and the pelvis, endochondral bone growth is also mildly impaired and causes a mild form of dwarfism [2, 4]. Delay in the eruption of permanent teeth and the presence of supernumerary teeth is a significant cause of morbidity [10] which in turn requires numerous oral surgeries and a long-term dental treatment [1]. As a result of the inadequate ossification of the contours of the embryonic vertebral arch, spinal deformities such as spina bifida, scoliosis, kyphosis/kyphoscoliosis, spondylolysis, spondylolisthesis, hemivertebra, posterior wedging of vertebrae, and cervical ribs may develop and these conditions may be seen together with the absence of the posterior thoracic vertebral arch or syringomyelia [1, 4, 10, 11]. Cleidocranial dysostosis has an estimated prevalence of 1/1,000,000 (Table 1). However, due to lack of diagnosis, its prevalence is estimated to be higher with no differences being reported between genders or ethnicities [1].

In order to better understand this rare skeletal dysplasia and the spinal deformities that accompany it, we present in this study the treatment outcomes in two patients with cleidocranial dysostosis and a review of the literature.

## 2. Case Report

We performed posterior fusion and instrumentation due to progressive scoliosis in two adolescent female patients diagnosed with cleidocranial dysostosis following genetic screening. Both patients had a positive family history of the condition. In addition to the spinal deformities, we thoroughly examined the concurrent orthopedic and dental problems of the patients. The mean age of the patients was 12 (range: 11 to 13) years at the time of surgery and the mean follow-up period was 11 (range: 6 to 16) years. The clinical and radiological outcomes were retrospectively evaluated (Table 2).

**Figure 1:**
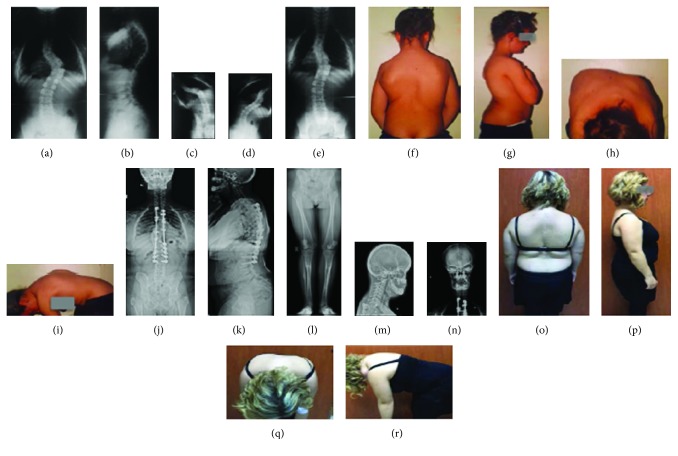
(a–i) CCD in an adolescent patient (B.Y.) accompanied by bilateral pseudoarthrosis of the clavicle, small face, protruding forehead, open anterior fontanelles, tooth eruption problems, short stature, and coxa vara. Posterior instrumentation and fusion was performed for scoliosis (posterior T2-L3 pedicle screw and fusion with hook construct). (j–r) Orthoroentgenograms and clinical appearance of the patient on postoperative 16th year.

**Figure 2:**
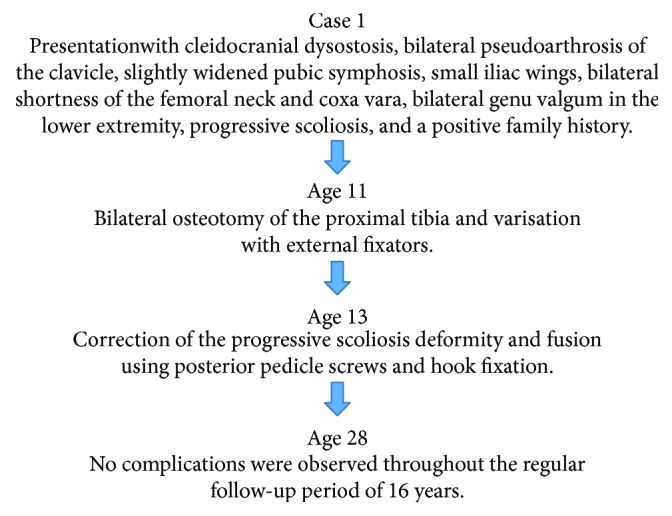
Timeline of the disorder and treatment for case 1.

In the first case (28-year-old female), the patient had the typical phenotypic characteristics of cleidocranial dysostosis (short stature, open anterior fontanelle, typical facial appearance, a wide and protruding forehead, and dental problems), bilateral pseudoarthrosis of the clavicle, slightly widened pubic symphysis, small iliac wings, bilateral shortness of the femoral neck and coxa vara, bilateral genu valgum in the lower extremity, progressive scoliosis, and a positive family history (in her father and grandmother) at presentation. Bilateral osteotomy of the proximal tibia and varisation with external fixators were performed to treat the gene valgum deformity (at the age of 11). Correction of the progressive scoliosis deformity and fusion was achieved using posterior pedicle screws and hook fixation (13 years). No complications were observed throughout the regular follow-up period of 16 years (Figures 1 and 2).

**Figure 3:**
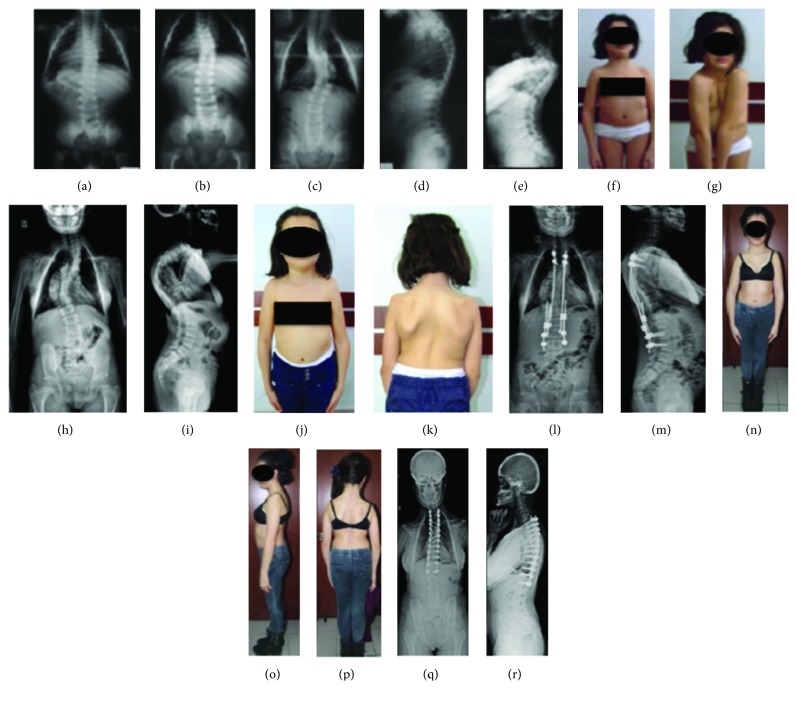
(a–g) Clavicular hypoplasia, widened pubic symphysis, bilateral coxa vara, progressive scoliosis, and kyphosis marked by growth and grade 1 spondylolisthesis in our female patient (E.T.) with CCD. Hypermobile shoulders typically coming close together before the chest, due to concurrent bilateral clavicular hypoplasia. (h–k) Radiological and clinical appearances of scoliosis and kyphosis. (l, m) Growing rod applied (from the posterior, between T2 and L3) at 11 years of age and later was lengthened two times in two years. (n–r) Clinical and radiological images from the second year follow-up of the 13-year-old patient, and Ponte osteotomy and fusion to the deformity apices (fixation with pedicle screws at all levels between T2 and L2 and Ponte osteotomy and fusion to the deformity apices).

**Figure 4:**
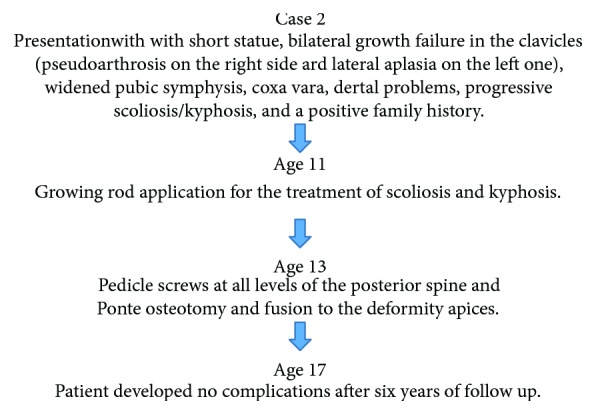
Timeline of the disorder and treatment for case 2.

The second case had short stature, bilateral growth failure in the clavicles (pseudoarthrosis on the right side and lateral aplasia on the left one), widened pubic symphysis, coxa vara, dental problems, progressive scoliosis/kyphosis, and a positive family history. At the age of 11, a growing rod was first applied on the patient for her scoliosis and kyphosis. The rod was lengthened two times in two years. At 13 years of age, the patient was applied pedicle screws at all levels of the posterior spine and Ponte osteotomy and fusion to the deformity apices. Six years after the first spinal surgery and four years after the fusion, the patient developed no complications. Adequate spinal correction and patient satisfaction were achieved (Figures 3 and 4).

## 3. Discussion

Cleidocranial dysostosis is a skeletal dysplasia inherited in an autosomal dominant manner and is characterized by abnormal formation of the endomembranous bone. The middle 1/3 of the clavicle, the cranium, and the pelvis are the most affected areas. Dental abnormalities are a common finding in patients [4]. Coxa vara is common due to metaphyseal disorders, and the deformity is usually of moderate degree and can self-heal with growth. In general, a genu varum deformity accompanies the condition and femoral and/or tibial osteotomy may be required [12].

Cleidocranial dysostosis is a condition inherited in an autosomal dominant manner in which 1/3 of the patients show spontaneous mutation and 2/3 show familial variation [4]. The responsible gene RUNX2 (Runt-related transcription factor 2) is a cloned gene located on the short arm of Chromosome 6 (6p21) [5–7]. RUNX2 activates the osteoblast differentiation as an osteoblastic-specific transcription factor and a regulator of osteoblast differentiation [6, 9]. It also controls the differentiation of precursor cells in osteoblasts. The cells secrete bone matrix and thus form a bone. In addition, RUNX2 plays a key role in the regulation of chondrocyte differentiation during endochondral bone formation. This new “principal gene” may explain the underlying mechanisms of bone formation in addition to the pathobiology of cleidocranial dysostosis [10].

**Figure 5:**
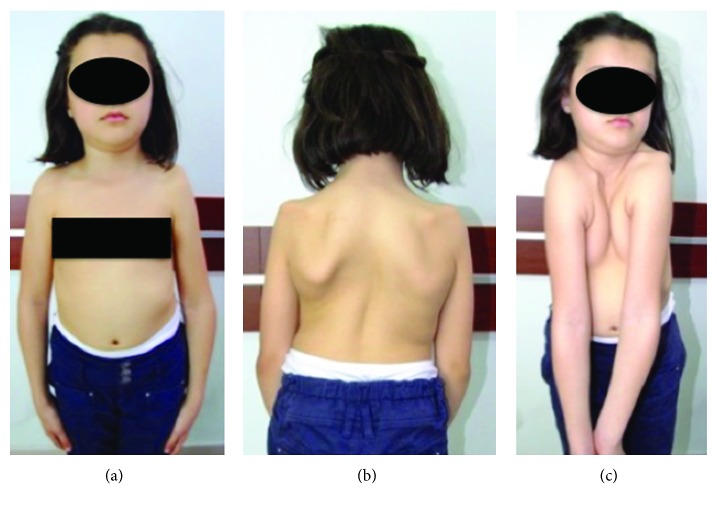
(a) Anterior and (b) posterior appearance of the clavicular winging in a 6.5-year-old girl with cleidocranial dysostosis. (c) Hypermobile shoulders typically coming close together before the chest, due to concurrent bilateral clavicular hypoplasia.

The condition typically manifests itself within the first two years of life. Although it has an estimated prevalence of 1/1,000,000, an incidence of 0.5 cases per 100,000 live births and more than 1000 cases until the year 2004 have been reported. Lachman described 38 cases based on his personal experience [3]. The affected children have a small face but a big head (the skull is bigger than usual but the face is smaller), the eyes are slightly wider, the palate is high and narrow, deciduous teeth emerge normally but the eruption of permanent teeth are delayed and imperfect and supernumerary teeth are present (65%) [2, 3], and the shoulders are low and the thorax looks narrow, thus leading to respiratory problems in the newborn [3, 13]. Anomalies in the sternum are due to abnormal intramembranous ossification and pectus excavatum is a prevailing condition [2]. One or both of the clavicles may show growth deficiencies and they may be totally absent [4]. The most common defect is the absence of the lateral end of the clavicle, followed by the growth failure of the middle 1/3 of the clavicle. The defect can be palpated. As a result of hypermobility in bilateral cases, the shoulders may come in contact with each other before the chest (Figure 5). The scapula may look smaller and the wings may be noticeable [2]. Patients with cleidocranial dysostosis are short, the mean height in adult males is between the 5th and 50th percentile of height for their age, whereas in females dwarfism is more apparent and the mean height is below the 5th percentile of height of their peers [10]. Progressive scoliosis or syringomyelia may be observed [3, 10, 11]. Susceptibility to Wilms tumor has been also reported [14].

**Figure 6:**
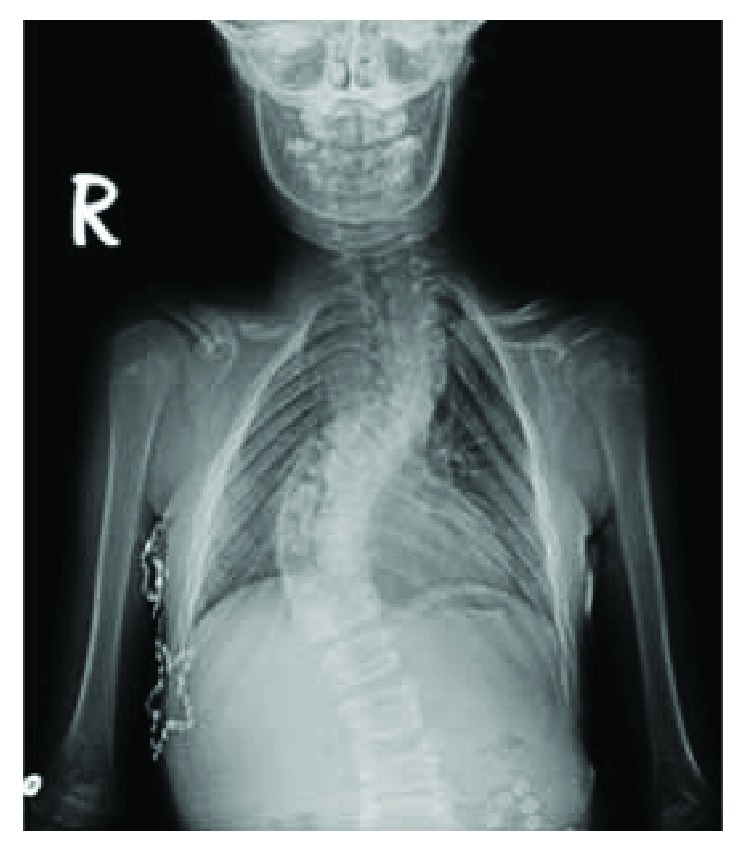
Radiograph of the same bilateral clavicular hypoplasia patient at age 11, showing poor growth of the middle 1/3 of the right clavicle and the absence of the lateral end of the left clavicle. Narrowing of the chest can be seen.

Orthopedic problems may include the absence of the clavicular end or hypoplasia of the muscles originating from or inserting into the clavicle, particularly the anterior part of the deltoid and sternocleidomastoid muscle. Hypoplasia and the absence of the clavicle can be clearly seen in the radiographs (Figure 6). The absence of the clavicle can be seen even in prenatal ultrasonography [15, 16]. The irritation of the brachial plexus is rare and may occur with pain and numbness. Excision of the clavicular fragment may lead to decompression of the brachial plexus. Scapular winging may be painful or symptomatic. Scapulothoracic arthrodesis has been described as the method of treatment for those cases [17].

**Figure 7:**
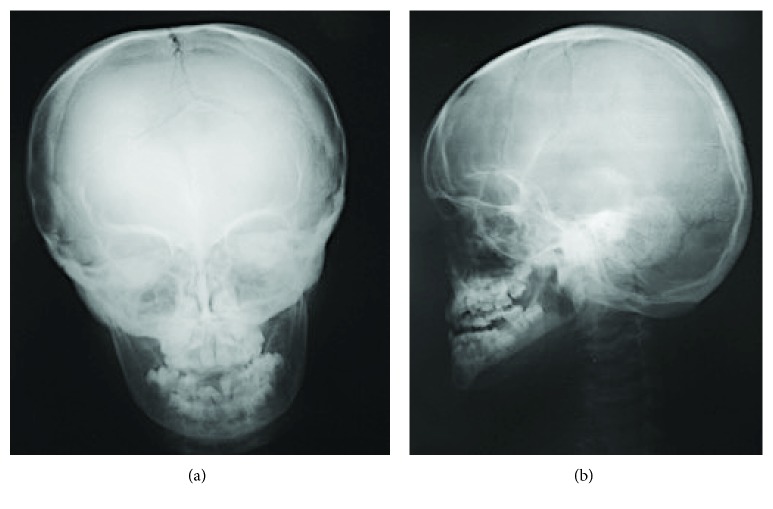
(a, b) Delayed closure of the sutures, widening of the anterior fontanelle, Wormian bones, sclerotic skull base, numerous supernumerary teeth, and malocclusion can be seen in the cranial radiographs (11 years).

Multiple Wormian bones and poor mineralization of the cranium are noticeable on cranial radiographs. Closure of the sutures is significantly delayed and the anterior fontanelle widens. In some patients, the anterior fontanelle never closes. Nasal, lacrimal, and malar bones may be hypoplastic or undeveloped, and the zygoma develops poorly. The maxilla may be small and the mandibular symphysis may be unfused (Figure 7) [2].

**Figure 8:**
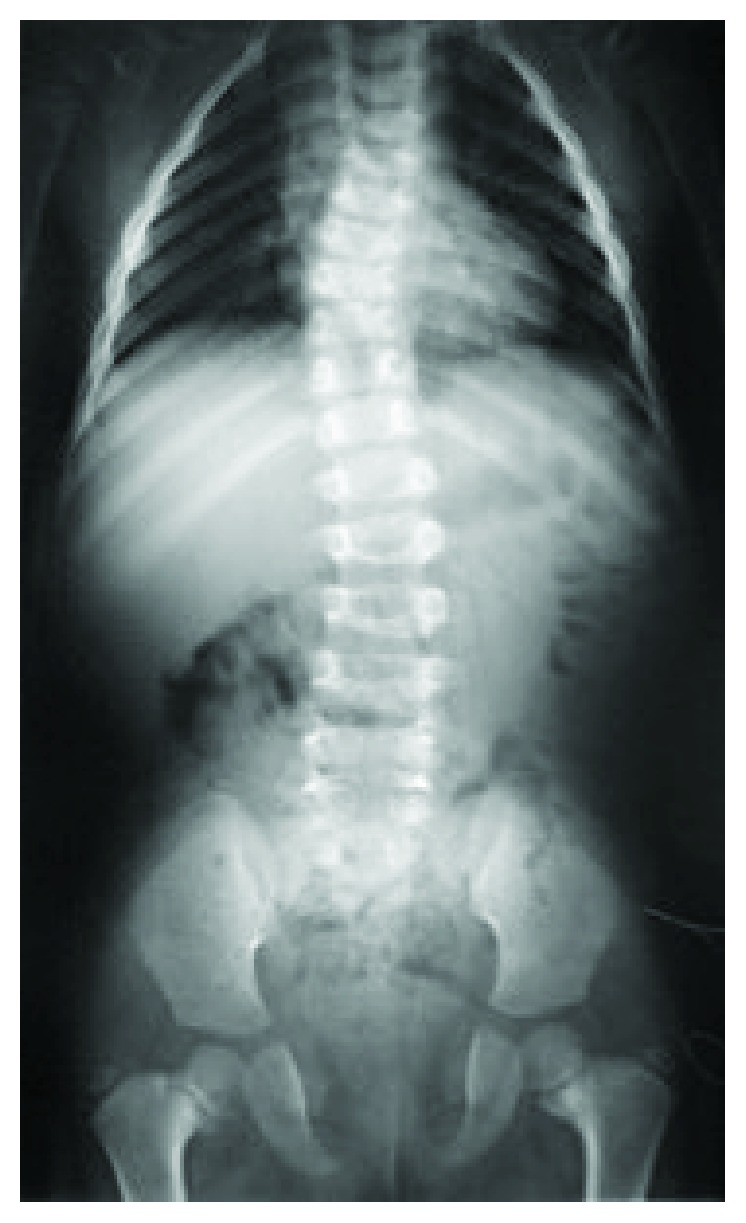
Widened pubic symphysis, bilateral coxa vara, and widened triradiate cartilage and sacroiliac joints can be seen in the pelvic radiographs (6.5 years).

The pelvis shows bilateral involvement. The pubic symphysis remains wide (Figure 8) [18]. Fusion may be incomplete or thin in ramus. The sacroiliac joint may be wider than usual. The iliac wings are small. Coxa vara may accompany cleidocranial dysostosis and the femoral neck is significantly short (Figure 8). Coxa vara is treated with the valgus osteotomy of the proximal femur. Indications for surgery are the same as those in developmental coxa vara (a head-neck angle of less than 90°, Hilgenreiner's epiphyseal angle of 60° or more, or progression of the deformity). Following osteotomy, the acetabular bone remodeling may be observed in young patients. Pelvic osteotomy is recommended to enhance the covering of the hip in older children [19]. Dislocation of the hip is rarely encountered [2].

Ossification is delayed in the carpal and tarsal bones. Terminal phalanges may be short, pointed, hypoplastic, or totally absent. Both the proximal and distal ends of the 2–5 metatarsals and metacarpals have epiphyses. The second metacarpal bone is usually long [2].

**Figure 9:**
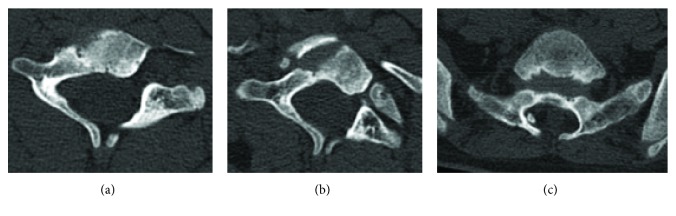
CT images showing spina bifida occulta in the (a) cervical, (b) thoracic, and (c) sacral spine of the patient (12 years).

**Figure 10:**
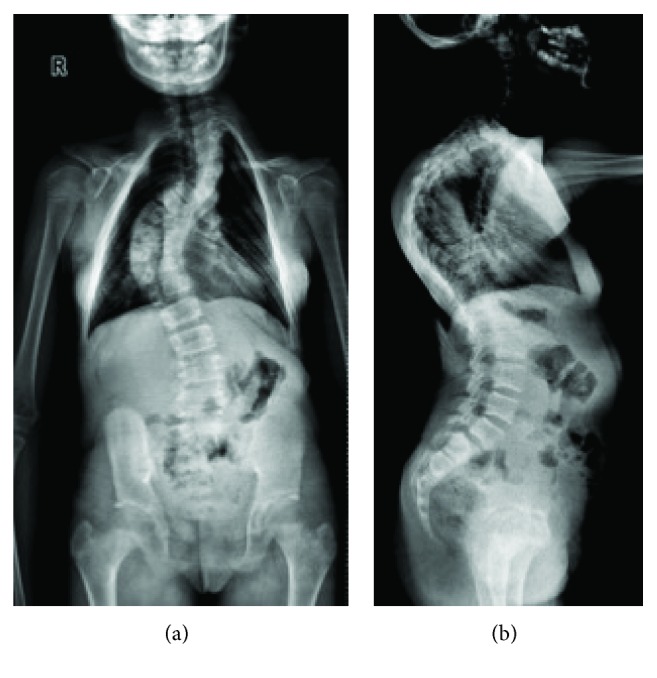
Progressive (a) scoliosis and (b) kyphosis in the spine (12 years).

**Figure 11:**
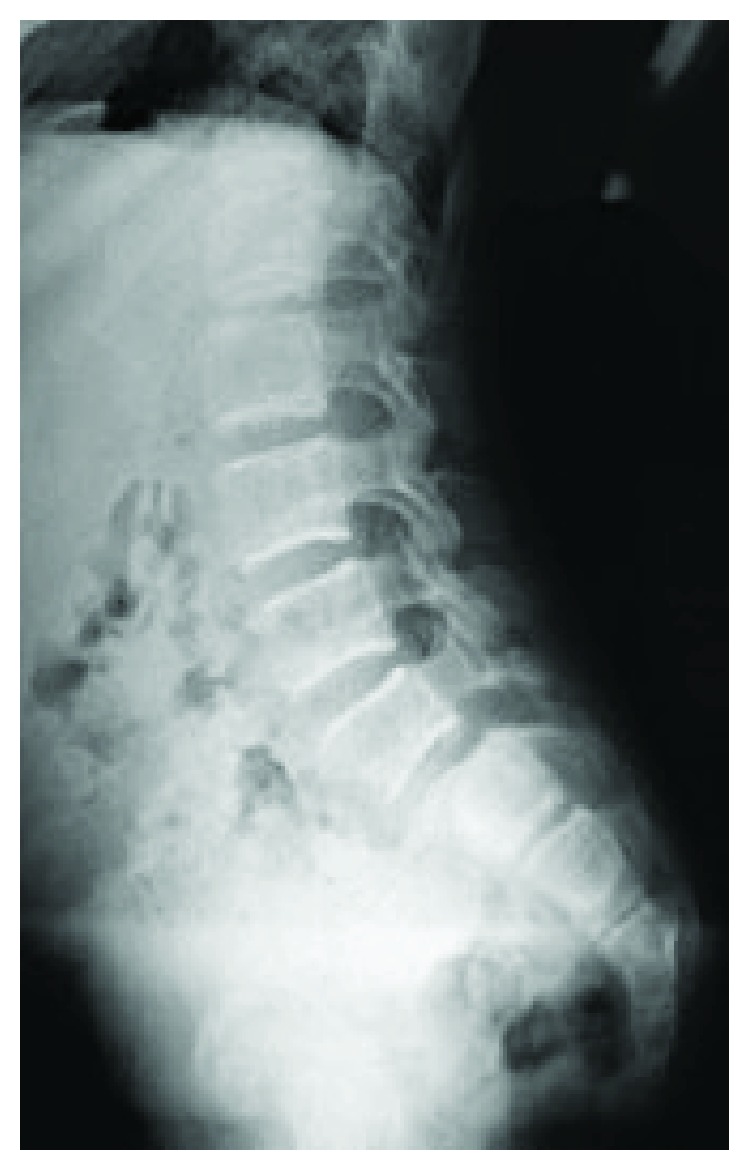
Concurrent lumbar spondylolysis (6.5 years).

Spina bifida occulta may develop in the thoracic and lumbar spine due to inadequate development of the posterior vertebral elements (Figure 9) [10], progressive scoliosis and kyphosis of the spine may be encountered (Figure 10) [9], and lumbar spondylolysis may occur in 24% of the patients concurrently (Figure 11) [10, 20]. The treatment of scoliosis in these patients is similar to that of idiopathic scoliosis (Figures 1 and 3) [2]. Syringomyelia has been also associated with the condition [21].

Cleidocranial dysostosis may be confused with pyknodysostosis due to the hypoplasia of the clavicle; however, osteosclerosis is not seen in patients with cleidocranial dysostosis but with pyknodysostosis [22, 23].

Differential diagnosis of congenital clavicle pseudoarthrosis may be necessary especially in the first years of life. Congenital clavicle pseudoarthrosis is almost always seen on the right side; only 10% of the patients show bilateral involvement. Dextrocardia may be encountered concurrently; the condition is congenital and the heart is usually pointed out toward the middle 1/3 of the clavicle [19].

Inadequate ossification of the contours of the embryonic vertebral arch may lead to spinal deformities such as spina bifida, scoliosis, kyphosis/kyphoscoliosis, hemivertebra and posterior wedging of vertebrae, and cervical ribs, and these conditions may be seen together with the absence of the posterior thoracic vertebral arch or syringomyelia [10, 21, 24]. A MRI scan of the spinal cord is recommended for syringomyelia and concurrent anomalies [21]. Spondylosis, spondylolisthesis, and spina bifida occulta may accompany the condition [10, 11]. Scoliosis may develop as a consequence to the imbalance of the shoulder girdle muscles and vertebral dysplasia [1, 11, 25]. Codsi et al. [24] suggested that the unilateral absence of the clavicle had a positive relationship with the rapid progression of scoliosis and that unilateral absence of the clavicle in immature children may lead to rapid progression of the curvature [1, 24]. In addition, the majority of the patients may encounter respiratory complications [1].

The surgical treatment in idiopathic scoliosis is also recommended for scoliosis. Codsi et al. performed posterior spinal instrumentation on a girl with rapid progression of scoliosis, hypoplasia of the posterior elements of the thoracic spine, and posterior fusion anomalies in C4–6. No complications were reported after five years of follow-up [24]. In a multicenter study, Cooper et al. [1] found significant increases in genu valgum, pes planus, sinus infections, upper respiratory tract problems, recurrent otitis media, and hearing loss of 90 individuals with cleidocranial dysostosis and their 56 next of kin, identified by genetic and dental examinations. The author also reported scoliosis in 16 patients (17%) from the cleidocranial dysostosis group and one patient from the control group (1.8%). Only three of the 16 patients had used braces; none of them required surgery, and the incidence of scoliosis was much higher than that in controls and the general population. Trigui et al. studied the different clinical aspects of cleidocranial dysostosis and orthopedic problems in two cases and reported that dental anomalies, coxa vara, and scoliosis needed regular follow-up and, in case of worsening of the symptoms, these problems should be treated [26]. Al Kaissi et al. [11] observed progressive scoliosis and kyphosis in five of seven patients with cleidocranial dysostosis and defined spinal deformities as problems leading to progressive and major orthopedic problems. The authors also stated that the spinal deformity may progress in continuation of the cartilaginous spinal structure. Injuries to the craniocervical region in cleidocranial dysostosis patients can lead to a wide range of complications from nondisplaced avulsion fractures of the occipital condyle to complete atlanto-occipital or atlanto-axial dislocations which may lead to morbid or fatal outcomes. Therefore, a thorough examination and evaluation of the patients with scoliosis deformity has been recommended [11]. In their retrospective study of 13 patients with deformities associated with rare disorders, Soultanis et al. performed posterior instrumentation and fusion on an 18-year-old male cleidocranial dysostosis patient with a rigid thoracic curve (85°) and spina bifida in the lower cervical and superior thoracic spine and reported that the patient was stable after seven years of follow-up [27]. Kobayashi et al. [28] published the clinical course and treatment outcomes of a 27-year-old female patient with cleidocranial dysostosis and spastic myelopathy due to atlanto-axial subluxation. The patient was operated two times for cervical myelopathy and atlanto-axial subluxation and had undergone a laminectomy of the atlas and C1-C2 fusion via a transpharyngeal approach and cervico-occipital fusion using Luque rod systems. The patient developed solid fusion at the postoperative seventh month and the MRI scan confirmed that the spinal cord was no longer decompressed; however, atrophy was still present. After two years of follow-up, the patient showed no neurological progress and still had spasticity. Although myelopathy due to atlanto-axial subluxation is rarely encountered in patients, it should be kept in mind during the follow-up and treatment of this disorder.

The limitation of our study was that we were able to evaluate only two patients. As the condition is a rare one, further multicenter studies are required in order to perform a more comprehensive assessment.

In conclusion, cleidocranial dysostosis may lead to complications such as scoliosis and kyphosis concurrent with various orthopedic involvements due to skeletal dysplasia. Since concurrent spinal deformities are of progressive nature, as in our cases, surgical treatment may be necessary. In addition to other orthopedic problems, possible accompanying complications such as atlanto-axial subluxation, myelopathy, syringomyelia, congenital spine deformities, spondylosis and spondylolisthesis, respiratory problems, and postoperative complications should be kept in mind while planning for the treatment of scoliosis and kyphosis. Through lengthening using growth-friendly systems (growing rod) in patients, like ours, with an early onset of symptoms, and once the spinal growth is complete, performing posterior instrumentation and fusion, as we did in both our cases, will yield successful results with no complications in the middle and long term. Further multicenter studies with more comprehensive assessments are required to find solutions to spinal problems related to this rare skeletal dysplasia.

## Figures and Tables

**Table 1:** Distinguishing characteristics of cleidocranial dysostosis.

Distinguishing characteristics of cleidocranial dysostosis	
Heredity	Autosomal dominant
Responsible gene and chromosome	RUNX2 gene/6p21 chromosome
Stature	Shortness of stature (K > E)
Prevalence	<1 million
Appearance of the face	Protruding frontal and parietal bones, depressed nasal bridge Tooth eruption problems Incomplete fusion of the mandibular symphysis Small face Slightly widened eyes High and narrow palate
Skull	Wormian bones Open fontanelles in children No cranial nerve palsy Wide head
Clavicle	Partially present or totally absent Irritation of the brachial plexus irritation (rare)
Scapula	Small, wings may be noticeable Winging may be painful or symptomatic
Thorax, sternum and shoulders	Narrow thorax and pectus excavatum Low shoulders Sternum anomalies
Hands and feet	Delayed ossification in the carpal and tarsal bones Terminal phalanges are short, pointed, hypoplastic, or absent Presence of epiphyses on both the proximal and distal ends of the 2–5 metatarsals and metacarpals Second metacarpal bone is usually long
The pelvis and hips	Wide pubic symphysis Wide triradiate cartilage and sacroiliac joints Small iliac wings Coxa vara, short femoral neck Hip dysplasia (rare)
The spine and intraspinal structures	Spina bifida occulta (thoracic and lumbar) Scoliosis Lumbar spondylolysis (24%) and spondylolisthesis Hemi vertebrae, posterior wedging Syringomyelia Myelopathy due to atlanto-axial subluxation (rare)
Other conditions	Susceptibility to Wilms tumor

**Table 2:** Distinguishing features in cleidocranial dysostosis patients.

Initials	Age (yrs) Sex	Follow-up (yrs)	Clinical and radiological findings	Treatment	Scoliosis (°)		Kyphosis (°)		Complication
					Preop	Follow-up	Preop	Follow-up	
BY	13 (F)	16	Typical appearance in the face and head, tooth eruption problems, short stature, scoliosis, coxa vara, wide pubic symphysis, deformity of the lower extremity, clavicular hypoplasia, osteopenia	Posterior pedicle screw fixation and fusion with hook constructs of T2-L3	52/60	37/38	50	37	—
ET	11 (F)	6	Small face and anteriorly protruding forehead, clavicular hypoplasia, tooth problems, kyphoscoliosis, wide pubic symphysis, coxa vara	Posterior T3-L3 growing rod, following gradual lengthening Posterior pedicle screw fixation of T2-L2, Ponte osteotomy, fusion	74	19	70	34	PJK (25°) development following growing rod application. Fixed with posterior fusion of the next level above
